# Etiology, Pathology and Treatment of Pellagra

**Published:** 1911-11

**Authors:** Geo. C. Mizell

**Affiliations:** Gastro-Enterologist to Wesley Hospital; Formerly Associate Professor of Physiology and Gastro-Enterology of Atlanta College of Physicians and Surgeons


### Journal-Record of Medicine

Successor to Atlanta Medical and Surgical Journal, Established 1855, and Southern Medical Record, Established 1870.

OWNED BY THE ATLANTA MEDICAL JOURNAL CO.

**Published Monthly**

*Official Organ Fulton County Medical Society, State Examining Board, Presbyterian Hospital, Atlanta, Birmingham and Atlantic Railroad Surgeons' Association, Chattahoochee Valley Medical and Surgical Association, Etc.*

EDGAR G. BALLENGER., M. D., Editor.
BERNARD WOLFF, M. D., Supervising Editor.
A. W. STIRLING, M. D., C. M., D. P. H., J. S. HURT, B. Ph., M. D.
GEO. M. NILES, M. D., W. J. LOVE, M. D., (Ala.); Associate Editors.
E. W. ALLEN, Business Manager.

COLLABORATORS

Dr. W. F. WESTMORLAND, General Surgery.
F. W. McRAE, M. D., Abdominal Surgery.
H. F. HARRIS, M. D., Pathology and Bacteriology.
MICHAEL HOKE, M. D., Orthopedic Surgery.
CYRUS W. STRICKLER, M. D., Legal Medicine and Medical Legislation.
E. C. DAVIS, A. B., M. D., Obstetrics.
E. G. JONES, A. B., M. D., Gynecology.
R. T. DORSEY, Jr., B. S. M. D., Medicine.
L. M. GAINES, A. B., M. D., Internal Medicine.
J. N. LeCONTE, M. D., Disease of the Stomach and Intestines.
L. B. CLARKE, M. D., Pediatrics.
EDGAR PAULIN, M. D., Opsonic Medicine.
THEODORE TOEPEL, M. D., Mechano Therapy.
R. R. DALY, M. D., Medical Society.
A. W. STIRLING, M. D., etc., Diseases of the Eye, Ear, Nose and Throat.
BERNARD WOLFF, M. D., Diseases of the Skin.
E. G. BALLENGER, M. D., Diseases of the Genito-Urinary Organs.

VOL. LVIII. NOVEMBER 1911 No. 8.

### ETIOLOGY, PATHOLOGY AND TREATMENT OF PELLAGRA.

BY GEO. C. MIZELL, M. D.

*Gastro-Enterologist to Wesley Hospital; Formerly Associate Professor of Physiology and Gastro-Enterology of Atlanta College of Physicians and Surgeons.*

It is the purpose of these articles to show that the consuming of linolin is the cause and the only cause of Pellagra.

Before going further with the discussion of the factors involved it will be well to summarize the argument that has already been advanced. Given below are the reasons so far brought out that linolin should be considered the cause of Pellagra.

1. The introduction of cotton seed oil in the United States was followed by the development of Pellagra.

2. In France, the first seed-crushing machine was established in Marseilles in 1817. Twelve years later Pellagra was reported to the Royal Society of Medicine of Bordeaux.

3. In Italy, Pellagra prevails most extensively in the northern portion where the poor have for centuries consumed as comestible fat oil of walnut.

4. Pellagra is confined to nations that eat oil containing linolin in large percentage.

5. All nations that consume much linolin are affected by Pellagra except as modified by climate.

6. The reports of the experiments with spoiled corn tend to show that the symptoms produced were due to the oil or oxidation products of the oil of corn.

7. These oils are poisonous, as is shown by their effects when fed to animals beyond a certain point.

8. The oxidation products of linolin are capable of producing the symptoms.

9. The effects of food containing linolin when fed to animals in large amounts resemble Pellagra, in that animals can consume with safety more oil in winter than in summer; in that it causes extreme weakness; in that it causes abortions, miscarriages and still-births; in that it causes diarrhoea and constipation. Some animals have mental disturbances. The eyes may be affected. Animals fed on food containing much linolin cannot stand the hot sun or do much work in summer. (That linolin is the injurious agent is evident because it is the only substance present in quantity that is not common to other stock feed and that cows of all stock can eat cotton seed meal and hulls in sufficient amount to maintain weight and only when they are giving milk. It is conclusive that cows are protected by lactation, that is, eliminating linolin as butter fat).

10. Linolin (consumed in large amount) is not all changed in digestion and constructive metabolism into normal animal fat (olein, palmitin, stearin) but is to some extent deposited as linolin.

Linolin, as has been demonstrated, is deposited in greatest proportion under the skin where the dermatitis is most marked, viz., in the extremities.

Each proposition stated above, except 5, 6, and 8, has been fully discussed in these pages.

Regarding the eating of cotton seed oil in the United States, it needs to be pointed out that the first products were mixtures of animal fats and cotton seed oil. These were eaten by very few people at first and only until the last few years have the cooking oils come into extensive use. There may be a few lards on the market sold for pure lard which contain a large per cent. of cotton seed oil, also many butter substitutes contain some cotton seed oil.

When the article showing the extent of oil consumption was written for the September issue of the Journal there was very little information on hand bearing on the kind of oil consumed in Italy. Information is now available to show that walnut oil has long been extensively eaten in vegetables and as dressing for fish. Vegetables, fish, walnut oil and polenta are the staple articles of food in the regions affected by Pellagra.

Statement 6 will be discussed later.

Statement 5, pertaining to the effects of climate, and statement 8, which has been in part discussed, will now be considered.

Pellagra has been geographically confined to tropical and semi-tropical regions. It is known that other than these regions consume linolin, hence if linolin is the agent that produces the disease there are some factors that limit the operation of the cause in other regions.

It is proposed that these factors are the color of the skin and sunlight. Bear in mind that we are discussing Pellagra with all of the characteristic symptoms and not an undeveloped or atypical case. It is being suggested that sprue is Pellagra sine Pellagra. A native Italian now resident in Atlanta commented to me upon the sore mouth prevalent in the mountains of Northern Italy. Samborn's ability to locate the area in which Pellagra was contracted was probably due to the fact that the farm hands develop the dermatitis only when they go to the fields in the spring and fall and are exposed to the sunlight. It is likely that sprue and Pellagra, as well as some other disease now considered as different diseases, will be shown to have the same origin.

Many cases have come under my observation that presented all of the features of sprue, according to Manson. Some of these cases have had dermatitis, while others were entirely free. Another class of cases correspond with the clinical pictures described by Manson as occurring in beri-beri. I have seen several patients, some without and some with dermatitis, who present all the features of dropsical beri-beri, and others who seem to be suffering with the paraplegic form.

All of these cases have been consumers of cotton seed oil and the most successful treatment has been the same as that given for Pellagra. Fortunately, in only two instances has there been a fatal issue among those cases which presented symptoms that would warrant a diagnosis of beri-beri according to Manson, hence the typical termination described by him has not often been observed.

The complex symptomatology of pellagra is not approached by any other disease, and so far as I know explanation of the factors that determine the course of the disease has not yet been suggested. These factors may be tabulated as follows:

| | |
|---|---|
| | Continuous consumption |
| Linolin | Intermittent consumption |
| | Amount consumed |
| | Intense |
| Sunlight: | Moderate |
| | Negative |
| | Blonde |
| Color of Skin | Brunette |
| | Black |
| | Disposition to accumulate fat |
| Individual Characteristics: | Disposition to leanness |
| | General diet |
| | Development per se |
| Precipitating cause: | Which may be any abnormal condition which increase physiological fat consumption. |

Complicating diseases or conditons, which are legion in adult life.

It is important to have a thorough understanding of some of the physical and chemical properties of the fats and oils in order to apply what is to follow on the action of light and color of skin.

(The information relating to the chemistry of fats and oils is obtained from the works of A. C. Wright, Alder Wright and Ainsworth Mitchell, Brant, Gill and Lewkowistch.)

### Physical Properties.

Physiologists speak of Olein, Palmitin and Stearin as the normal fats. It is however, shown that animal fat takes on somewhat the same nature as that upon which the animal is fed. Dogs fed upon linseed oil yield a fat that smells of linseed oil; hogs fed on dried fish yield fat that has a fishy taste. Many animal fats contain linolin when the animals are fed corn or oil cake. Fat from an Eskimo is influenced by blubber oil, while that from Polynesians, living chiefly on cocoanuts, yield fat resembling somewhat cocoanut oil. Lard from a hog fed on cotton seed oil cake gives as strong a test as a mixture of lard with thirty per cent. cotton seed oil.

The fats are distributed throughout the body, being present in all the fluids and solids except the eyelids and urine. Raymond gives the percentage of fats in the various tissues as:

| | | | |
|---|---|---|---|
| Sweat | .001 | Cartilage | 1.3 |
| Vitreous humor | .002 | Bone | 1.4 |
| Saliva | .02 | Crystaline lense | 2.0 |
| Lymph | .05 | Muscles | 3.3 |
| Synovia | .06 | Liver | 2.4 |
| Liquor Amnii | .06 | Hair | 4.2 |
| Chyle | .2 | Brain | 8.0 |
| Mucous | .3 | Nerves | 22.1 |
| Blood | .4 | Adipose tissue | 82.7 |
| Bile | 1.4 | Marrow | 96.0 |
| Milk | 4.3 | | |

Some of the offices which fat subserves in the body are: it is a lubricant, as in the hair, skin and synovial fluid; it is a reservoir of fuel when the food is cut off; thus in wasting diseases the deposited fat is absorbed and supplies nutrition; it is a source of heat and energy through its oxidation.

The three fats mentioned above are of different fluidity, that is, they have a different melting point. They are present in such proportion as to render human fat fluid at the temperature of the body. The melting point of the fat varies with the region in which it is located. By the melting point it is possible to determine from what part of the body a specimen of fat has been obtained. This is shown by the following table:

LARDS FROM DIFFERENT PARTS OF HOGS.

| Fat from: | Melting point: | Observer: |
|---|---|---|
| Foot (hoof) | 35.1°C | Wiley |
| Leg | 42.5°C | " |
| Ham | 42.5°C | " |
| Back | 44.5°C | Spaeth |
| Kidney | 48.2°C | " |
| Leaf | 44.0°C | " |

The consistency (melting oint) of a mixture of fats is dependent upon the proportion of the various fats present in the specimen.

In general it may be stated that the proportion of the more liquid fats diminish as the position approaches the warmer parts of the body, therefore the fat in the extremities has the largest per cent. of liquid fat and the internal fat contains the largest per cent. of solid fats. The melting point of some of the various fats and oils is shown below.

| | Melting point °C: |
|---|---|
| Beachnut oil | —16.5 |
| Cotton seed oil | — 2. |
| Walnut oil | —28. |
| Poppy oil | —16. to —18. |
| Sesame oil | — 5. |
| Sunflower seed oil | —16. |
| Butter | 20. to 30. |
| Olive Oil | 2. to 4. |
| Cocoanut oil | 20. to 20.5 |

| | |
|---|---|
| Sheep tallow | 47. |
| Ox Tallow | 43. |
| Horse fat | 20. to 41.8 |
| Hare fat | 35. to 41.0 |
| Human fat | 17.5 |

The low melting point of the first six oils is due to the large percentage of linolin which is present.

The points in the above which interest us here and which are to be borne in mind are:

1. The wide distribution of fat in the body.

2. The melting point of fat varies according to the position.

3. A fat is to some extent deposited in the tissues of an animal in the original chemical form of the fat consumed.

While the fats and oils are all composed of carbon, hydrogen and oxygen and have some properties in common, they also possess physical and chemical properties which vary greatly in degree and the products of oxidation are not always the same. The classes of fat that pertain to the subject are those edible fats containing linolin and those containing none or an insignificant amount of linolin. The former are the drying and semi-drying vegetable fats and the latter the non-drying vegetable and animal fats. It will be most convenient to compare the chemical and physical properties of linolin, the characteristic constituent of the drying oils, to olein, a representative of the non-drying group of fats to which it is most closely related. The melting point of linolin and olein has been pointed out above, and this is the only physical property of importance.

### Chemical Properties.

The chemical properties of a fat or oil are dependent upon the nature of the fatty acid, so a consideration of these properties mainly pertains to the properties of the fatty acid.

All fats possess the property of absorbing oxygen from the air. Fats containing linolin absorb oxygen much more rapidly than the non-drying fats.

This action is seen in the drying of paints and in the thickening of various oils when they are exposed to air. Oxygen is absorbed at ordinary temperature, but absorption is much increased by heat or the presence of an oxidizing agent such as permanganate solution, hydrozen peroxide, etc.

The presence of light produces a marked increase in the spontaneous oxidation of fats which takes place at ordinary temperature. Specimens kept in the dark for six months show little or no change, whether in tightly corked or open bottles, and whether left undisturbed or shaken daily so as to aerate them. Similar secimens exposed to sunlight during the same time showed marked changes even when kept undisturbed in corked bottles. Evidence of the action of light is found in the table given below:

VARIATION IN SPECIFIC GRAVITY:

| | Original value practically unchanged in the dark: | Value after six months' exposure to sunlight. Undisturbed: Corked: | Agitated daily: Uncorked: |
|---|---|---|---|
| Olive Oil | 0.9168 | 0.9185 | 0.9246 |
| Cotton seed oil | 0.9225 | 0.9236 | 0.9320 |

VARIATION IN IODINE ABSORPTIONS

| | Original value practically unchanged in the dark: | Value after six months' exposure to sunlight. Undisturbed: Corked: | Agitated daily: Uncorked: |
|---|---|---|---|
| Olive Oil | 83.16 | 82.64 | 78.24 |
| Cotton seed oil | 106.84 | 106.40 | 100.12 |

VARIATION IN HEAT EVOLUTION WITH SULPHURIC ACID.

| | Values after keeping six months In the dark: Undisturbed: Corked: | Agitated daily: Uncorked: | In the light: Undisturbed Corked: | Agitated daily Uncorked: |
|---|---|---|---|---|
| Olive Oil | 44°C | 43.5° | 47. C | 67. C |
| Cotton seed oil | 75 5 | 76.5 | 76.5 | 100 |

The action of light and air upon fats causes an increase in fatty acids and other chemical changes.

No changes are produced by the action of light when oxygen is entirely excluded. The chemical changes according to Spaeth are summarized as follows:

1. The unsaturated acids are first attacked with the formation of acids containing less carbon, and subsequently aldehydic substances and hydroxy acids are produced.

2. In the course of oxidation and formation of free acids the amount of volatile acids is considerably increased.

3. All fatty acids present contribute to the formation of free fatty acids.

4. As the oxidation proceeds the power of absorbing iodine correspondingly decreases, owing to the above-mentioned oxidation, and decomposition of the unsaturated fatty acids and their polymerisation. Fats thus oxidized have considerably higher refractive index than normal fats, which must be attributed to polymerisation of the fatty acids.

5. In general, fats thus oxidized show a higher melting point than fresh fats. Mjoen's experiments show that the changes which take place in fats in the light and in the absence of sunlight are of a distinctively different character. Nagel has identied in these oxidized fats substances belonging to the following class of compounds: (1) free fatty acids; (2) hydroxy fatty acids; (3) lactones and anhydrides of fatty acids; (4) alcohols; (5) esters of various fatty acids; (6) aledhydes; (7) acetals, and (8) terenes.

Scala has also identified many of the above oxidation products.

He found in rancid olive oil and lard formic, butyric, caproic, heptylic and nonylic acids and aldehydes.

In his opinion all the aldehydes and the corresponding acids are produced from olein and oleic acid, while the absence of formaldehyde leads him to believe that the formic acid is due to the slow oxidation of glycerol, which can yield formic acid, formaldehyde being an intermediate product.

Darkin observed that by using mild oxidizing agents (such as hydrogen peroxide) all fatty acids, from formic acid up to and including stearic acid, can be oxidized at comparatively low temperatures, whereby a series of intermediate products are formed. Lewkowitsch suggests that this may throw some light on the biological oxidation of *fats and fatty acids in vivo.*

The various oils and fats exhibit different degrees of susceptibility to the action of light and air (ozygen), the solid fats undergo change most slowly; the liquid non-drying oils undergo change more readily, and the drying oils most readily. The rapidity with which this last class of oils undergo chemical change has offered great obstacles to the investigation of the oxidation products. The rate and amount of oxygen absorption (oxidation) is determined by exposing the oils to air and light and noting the difference in weight before and after exposure. The difference in weight and develoment of fatty acid is shown in the following table by Wright and Mitchell:

PERCENTAGE INCREASE IN WEIGHT

| | Of Oil after 2 days: | 7 days: | Of Fatty Acids after 8 days: |
|---|---|---|---|
| Walnut oil | 7.9 | | 6.0 |
| Poppy oil | 6.8 | | 3.7 |
| Cotton seed oil | 5.9 | | 0.8 |
| Sesame Oil | nil | 2.4 | 2.0 |
| Olive oil | nil | 1.7 | 0.7 |
| Arachis oil | nil | 1.8 | 1.3 |

The information furnished by the above tables simply shows the process of absorption. It will be noticed that the changes in olive oil in the dark are greater than those in cotton seed oil, and that the changes in cotton seed oil in the light exceed those in olive oil. The last table shows that the rate of oxidation is much more rapid in the drying than in the non-drying oils.

Cloes' investigations in regard to the effects of light in promoting oxidation are also interesting. He shows that the changes are very slow in the dark, though in some oils and fats the change is in time as great as under light.

The effects of different colored rays are also very different. The change is at first most rapid under the white light, less so under blue, and much less under red, yellow, and least of all under green, but in time blue overtakes and passes the white and at length all are about equal in effect. Another factor which may be of importance is that the introduction of a small amount of oil which has been exposed to air into the unchanged oil greatly accelerates the oxidation. This fact indicates that the change, slow at first, is accelerated once oxidation is begun.

The rapid oxidation of fat may produce enough glycerol to develop a pathological condition. Marked toxic action of glycerol is demonstrated when it is injected into the blood of animals, producing nervous symptoms and death. It is also laxative. It has been pointed out above that in the spontaneous oxidation of oils and fat formic acid may be produced from glycerol formaldehyde probably being an intermediate product.

From the above may it not be claimed that the oxidation products of fats are capable of producing the symptoms which develop in Pellagra? These oxidation products are all more or less physiological and it is only when an increased production through oxidation occurs that the pathological condition is developed. Chemists have found it difficult to study the oxidation products of oils and fats, especially linolin; in fact, the obstacles are so great that very little is known of their properties.

The oxidation products which are suspected of producing the pathological condition termed Pellagra are: Fatty acids, Aldehydes, Glycerine, Formaldehyde and Formic acid, Sativic acid being an unknown quantity.

### Physiological and Pathological Chemistry.

Beginning with the entrance of fat in the food, we will trace it through the body to its final disposition. As we proceed, bear in mind that Stearin, Palmitin, Olein and Linolin oxidize spontaneous; this property is increased in alkaline solution and in the presence of oxygen; also that Linolin oxidizes much more rapidly than the other fats named above.

The fats of the food are absorbed in the form of fatty acids, soaps, i. e., fatty acids in combination with alkali, glycerine and emulsion. It is thought that only a small proportion, if any, of the fat is absorbed as emulsified fat. The fatty acids after entering the walls of the absorbing organs of the small intestines are united with glycerine, which has been absorbed along with it, forming neutral fat. The nature of this neutral fat is governed by the kind of fat taken as food and therefore has the same properties. Passing into the blood as neutral fat it is carried throughout the body, some being oxidized immediately and a portion being deposited in the various tissues as neutral fat. The accumulation of fat in the body is favored by the chemical constitution which renders fats more difficult to oxidize than carbohydrates and proteids.

Although carbohydrates may be converted into fat and stored as such in the body it is thought that the ingested fat is more important in the human animal. After it enters the blood fats are in an alkaline solution containing the oxidizing agent haemoglobin and after it is deposited in the tissues the same conditions obtain to a less extent, i. e., the association is not so intimate. When the fat is deposited directly under the skin, oxidation is necessarily favored by the action of light.

When the ingested fat is composed of stearin, palmitin and olein, there is a normal rate of oxidation taking place at all times in the tissues and when the ingested fat exceeds the needs of the organs and tissues it is stored as stearin, palmitin and olein in the regions common to fatty deposits. It necessarily follows that should linolin be substituted for stearin, palmitin and olein, a greater per cent. will be oxidized, if not in the tissues before it is deposited, certainly in the blood. The continuous formation of fatty acids in the blood would thereby be increased, which would result in an excess of the salts of fatty acids and glycerine. This excess of salts of acids and glycerine in the body is further increased by the rapid oxidation of the stored linolin. The fatty acids contained in olein, palmitin and stearin are among the most stable organic compounds and are attacked only by very vigorous agents which is in great contrast to the easily oxidizable linolic acid.

The ease with which linolin undergoes oxidation closely approaches this property in most of the acids of the lower fatty acid group. Herter relates the pathological chemistry of some of these lower fatty acids and his observations are quoted in full, as follows:

"Let me now say a word to you in reference to the organic acids which occur in the food. This subject has a certain relationship to the subject of alcohol, for as I mentioned to you, alcohol is probably oxidized to acetic acid in the organism before its final oxidaton to carbon dioxide and water. Some foods contain organic acds of the fatty acid series in considerable amounts. This is especially true of fruits and some vegetables. You should know something of the detsiny of these acids in the organism and of their effects on digestion, for there are many persons who are unable to take them without disturbances, and moreover some persons show very remarkable idiosyncrasies.

Like alcohol, the organic acids of the fatty acid series which I have just mentioned are oxidized in the body to carbon dioxide and water; and, as in the case of alcohol, this decomposition and oxidation is accompanied by the liberation of energy. These acids thus have a food value comparable with that of alcohol. Oxalic acid is burned in the body only to a limited extent.

"Most persons in health are able to take these various organic acids contained in fruit and vegetables without exhibiting any harmful effects from the moderate quantities which are introduced into the stomach in this way. The acids are absorbed as acid salts, and on being burned in the body become a source of energy. I am disposed to think that a not inconsiderable portion of the carbohydrates, or fats of the food, may be replaced by organic acids where the state of digestion permits abundant absorption.

"I recall very distinctly an expriment which I made some years ago with a young pig. The animal received the same amount of milk as other pigs in my laboratory, but in addition was induced to take from 50 to 100 c. c. daily of glacial acetic acid well diluted with water. The principal result of this experiment, which extended over many months, was that this particular pig grew much larger than his fellows. I was astonished to find at autopsy an enormous development of fat with a melting point lower than that of ordinary hog-fat. I believe this large development of fat was connected with the absorption and utilization of the large allowance of acetic acid, very little of which reappeared in the urine and faeces of the animal. The possibility of a synthesis of fat from acetic acid is suggested by ths experiment.

"But while the organic acids of fruits and vegetables are commonly utilized in the way I have just described, there are many persons in whom they create very decided disturbances. These persons usually show some of the indications of chronic gastritis, and are apt, I think, to secrete very little free hydrochloric acid during gastric digestion. Among the symptoms which follow the use of immoderate quantities of fruit are a sense of discomfort referred to the stomach, abdominal pain, sometimes amounting to colic, diarrhoea, or in some instances headache. That the symptoms are really due to the organic acids and not to other ingredients is rendered extremely probable by the fact that in certain patients the same symptoms can be elicited by giving separately malic or citric, or some other acid characteristic of a particular fruit.

"You will find that with many patients it is wise to withdraw fruits containing considerable quantities of organic acids or acid salts. In other cases it is not necessary to withdraw such food wholly from the dietary but to restrict it."

It is well known that fatty acids of the higher series (stearin, palmitin, olein), are to a degree irritating to the gastric mucous. That the more stable neutral fats are not free from changes in the stomach is evidenced by the rancid condition which is developed, especially in every slight retention. It is probable that a large amount of fatty acid is liberated in the stomach when linolin is the fat concerned. Irritation from the almost constant presence of irritating linolic acid will in great part account for the gastric disturbances which gradually develop and gradually increase in Pellagra. The presence of fatty acids in the stomach may in this way account for the action of oils in diminishing the secretion of hydyrochloric acid.

The increased production of fatty acid in the stomach and the rapid development of them in the intestines may produce some of the intestinal disturbances. Every uncomplicated case of pellagra under my observation gives a history of pre-existing constipation. It is reasonable to suppose that when easily digested linolin is substituted for the more stable fats the healthy condition of the intestines take up the fat readily and constipation favored. On the other hand, there may be impaired absorbing power, gradually brought about by the constant irritation of free linolic acid which on account of its peculiar chemical and physical properties is split into fatty acids and glycerine more rapidly than alkali is furnished by the secreting organs. It is evident to anyone who has observed the stools of advanced cases of Pellagra that obsorption is greatly hindered even in those cases where intestinal digestion is apparently good. As mentioned above, the constitution of linolin and the blood favors rapid oxida_ tion with the production of an excess of fatty acids, glycerine and salts of fatty acids, viz., soaps. As having some connection with the subject I quote Dr. F. X. Guraud, formerly chief of the Laboratory of the Medical Faculty of Paris, as follows:

"When the fats havae once entered the general circulation, they are accumulated in the liver, infiltrating it in a normal physiological manner.

"Thence they are gradually distributed throughout the tissues. It is easy, therefore, to understand how an excessive use of fat is able to choke the hepatic parenchyma.

"The fats play an important role in the process of nutrition, chiefly by their caloriferous power, for one gram of fat gives on an average double the number of calories that one gram of albumin and carbohydrates will produce. If the latter are superior in their action on the muscular system by force of the isoglucoric coefficient, the fats excel them in the struggle against the loss of heat. That is the reason why the inhabitants of cold climates consume such vast amounts of fatty substances. The fats do not only afford much fuel, but they restrict also the waste of energy, and influence the ecomonical principles within the system almost as much as do the ternary bodies. They also furnish, well nigh exclusively, the material for our reserve forces. Excessive alimentation modifies but little the surplus of nitrogen and sugar in the organism, because everything that is not burned up and consumed is converted into fat or adipose tissue. Hence it is that overfeeding becomes a danger. A surcharge of fat jams the organs, obstructs their movements, makes the circulation sluggish, and prepares the way to physical degeneracy. These dangers are frequently increased by cardiac localization

"Excretory. Fat is not discharged from the system in the same manner as water or carbonic acid. When it does not ferment it does not molest the kidneys. But the fatty acids which it produces are more troublesome, as they irritate the skin through which they are eliminated.

"Indications and Contraindications. In the normal individual the absorption of fat is the strongest protection against the loss of caloric force. In cold climates a larger amount of it is required, while in the warm countries the need for it is much restricted.

"In the temperate zones fatty foods should be used with moderation, especially during the heated term, if gastric troubles are to be forestalled. This precaution is indispensable in patients who require a calorific diet, such as consumptives and diabetics.

"It would appear that fat constitutes an eminent nourishment in acute disease in which caloric losses are so enormous; but, unfortunately, in these very conditions it is badly tolerated and therefore not absorbed, for which reason it has to be eschewed altogether."

As further evidence of the injurious effects of the oxidation products of fats on living cells I will mention the rritating action of rancid fats when applied to the skin. Another phenomena which may have some connection with the subject is well known relation of light to certain skin lesions, such as smallpox.

So far as I know, no explanation of the injurious action of light has been offered. It seems that the presence of fatty acid in pus and the action of light in favoring the development of injurious oxidation products may have some bearing upon the aggravation of these lesions when the patients are exposed to strong daylight.

I believe I can make the statement without fear of contradiction that there is no such thing as typical pellagrous dermatitis without exposure to strong daylight or perhaps without exposure to strong sunlight. There is one peculiar phenomenon connected with the disease. It is recorded somewhere that this has been demonstrated by exposing different areas of the skin and protecting others. Dr. Bass, of New Orleans, seems to be of the same opinion. Inmon, of Texas, states that pellagra on the plains is much more severe than in the lower altitudes. Not only are the skin lesions aggravated but also the constitutional symptoms, yet in the face of this several of our distinguished physicians persist in forcing these unfortunate patients out into the strong daylight and sunlight.

The relation of sunlight to the skin and constitutional symptoms is evident in a patient now under care and is so instructive that a little detial will be interesting. The patient, Mrs. S., widow, age 52, resident of Atlanta, not poverty stricken, had suffered with pain in gall bladder region and was operated on July 28th, 1911. Reacted poorly. Developed diarrhoea and sore mouth late in August. Attending Physician called in consultation a well-known Diagnostician. The result of the consultation was that Mrs. S. was placed out on a porch, facing south, exposed to the sun all day. People of this section will recall the clear sky and hot days of the past September. Mrs. S. had no dermatitis when she was confined to her room, yet after two weeks in the sun her hands had the appearance of having been baked. Nervous and mental symptoms had appeared; vertigo and pains in the extremities were distressing. At this time the patient was suffering to such an extent that she persuaded the family to move her into the house. Acting on the advice of the consultant she was placed in a room where she could be given a sunbath all over for a greater part of the day. The treatment and progress of the case was so unsatisfactory that the attending physician withdrew and I was called in on October 11th. The patient was immediately placed in a shaded room, and treatment begun. There has been daily improvement in all symptoms. As a result of the sun baths the skin over the whole body now presented a typical pellagrous dermatitis which is most severe on the hands.

Along the same line of thought it appears to me that the negro is much more immune to the skin lesions of pellagra than whites. We all know that all the etiological factors mentioned, Maize, Linolin, Mosquitoes, Simalium Reptans and Pecaurum, poverty, unsanitary habitation, etc., concern the negro to as great an extent as the whites, yet all the statistics and personal information on the subject go to show that dermatitis in the negro is greatly in the minority. This comparative freedom of the black skin from invasion may be explained by the obstruction offered to the chemical rays of the sun by the colored pigment. I am led to believe by the result of personal investigation that proportionately as many negroes have the disease, but rarely develop the dermatitis. My experience with negro patients is very limited, as none are treated in my private work and only a few, one negro to every fourteen whites, come for treatment to the public pellagra clinic. In these cases the disease was more advanced and the other symptoms more severe and fatal when the dermatitis appeared than in the white patients. I think for the same reason the different types of white people, blondes and brunettes, show a variation in the severity of the dermatitis. In fact, the blondes among my cases greatly outnumber the brunettes. Also those who tan do not suffer with as severe dermatitis or other symptoms as those who freckle.

Perhaps herein is a reason why animals would not develop the dermatitis if exposed to the cause of the disease. Dogs, rabbits, guinea pigs, and other animals are protected by hair from the chemical rays of the sun. The white rat is about the only animal having an unprotected appendage, the tail, which approaches the extremities of man.

A consideration of the linolin-bearing seed reveals a condition designed to exclude the light and air. Nature's Creator has not designed without purpose. It is evident that oils of this class are not intended to be used as a food for man to the practical exclusion of fats, which more closely approximate human fat. In all animals it appears that the character of the fat bears some relationship to the environment as regards light aad oxygen. Thus in animals, as in the vegetable kingdom, the fats occur as drying, semi-drying and non-drying according to environment. In water animals semi-drying and no-drying oils abound. In only two land animals, ice bear and rattlesnake, are drying oils found. Semi-drying fats are found in some wild animals to a limited extent, and in domestic animals only when present in the food given them.

401 Empire Life Bldg.

(To be Continued.)

